# Edaphic characterization and plant zonation in the Qaidam Basin, Tibetan Plateau

**DOI:** 10.1038/s41598-018-20163-0

**Published:** 2018-01-29

**Authors:** Xianjie Wang, Fanjing Kong, Weigang Kong, Wenning Xu

**Affiliations:** MLR Key Laboratory of Saline Lake Resources and Environments, Institute of Mineral Resources, Chinese Academy of Geological Sciences (CAGS), Beijing, 100037 China

## Abstract

This paper presents a study of edaphic characteristics and their relationship with plant distribution in the Qaidam Basin, Tibetan Plateau, and establishes a distribution model for plants in sandy gravel Gobi to dry salt lake areas. All of the communities in the study area were dominated by plants with strong saline-alkaline tolerance. In this area, salts appeared to migrate to the surface; the surface soil was striped, and the salt distribution varied from sandy gravel Gobi to dry salt lake areas. The salt composition mainly consisted of NaCl in the surface crust. In the subsurface layers, the salt composition was dominated by Ca^2+^, Cl^−^ and SO_4_^2−^. The type of vegetation at the study site can be divided into two categories: salt-tolerant vegetation and weakly salt-tolerant vegetation. The salt-tolerant vegetation is influenced by Na^+^, Cl^−^, and the salinity. The soil of these vegetation communities had a higher salt and Na^+^ concentration and a lower Ca^2+^ and K^+^ concentration. The weakly salt-tolerant vegetation is mainly affected by the Ca^2+^/Na^+^ and K^+^/Na^+^ ratios. Based on the above results, a vegetation distribution model for saline lakes on the inland plateau was established.

## Introduction

Plant zonation is a common characteristic of salt marshes worldwide^1^. In low-stress environments, competition is an important factor for plant distribution, but in harsh physical conditions, the tolerance of species to an extreme soil environment determines the plant distribution^2^. Therefore, the soil type, especially the soil salinity, is a major factor in plant zonati on^3–6^.

Solutes contributing to soil salinity include different ions, such as Na^+^, K^+^, Ca^2+^, Mg^2+^, Cl^−^, SO_4_^2−^, CO_3_^2−^, and HCO_3_^−^. The relative proportions of Ca^2+^, Na^+^, Mg^2+^ and K^+^ in the soil are considered to be critical factors for the development of plants in saline environments^7–10^. However, if the concentration of these ions is too high, the plant cells will suffer high osmotic pressure, which affects plant growth and development. Too much salt accumulation can also lead to a decrease in the osmotic pressure of plant roots, directly affecting the uptake of nutrients by roots, which can impair the growth of vegetation^11^. Furthermore, the concentrations of some ions do not necessarily increase when soil salinity increases. Therefore, determining the concentration of a specific ion or ion ratio is necessary when evaluating the relationship between soils and plants^1,6^. Álvarez Rogel *et al*. reported that the ion ratios of K^+^/Na^+^, Ca^2+^/Mg^2+^ and Ca^2+^/Na^+^ can best explain soil-vegetation relationships^1,6,12^.

Many studies have determined the relationships between soil salinity, soil moisture and plant zonation on coast marshes^1,6,12–17^. For instance, the temporal gradients and topography of the salt marshes in Spain have been studied. A relationship model between soil and vegetation was established by analysing the correlation between the soil environment and specific vegetation types, allowing the vegetation to be used as an indicator of soil type^1,6,12,14^. However, few studies on vegetation response to soils have been conducted in salt lakes. This paper focuses on the vegetation and soil characteristics on watershed of salt lake and a vegetation distribution model of saline lakes on an inland plateau is put forward.

There are many saline lakes in China; therefore, the total salt marsh area is large. The Qaidam Basin is located in the northeast part of the Qinghai-Tibetan Plateau, which is the largest and highest plateau in the world. The basin is surrounded by the Kunlun Mountains, Altun Mountains and Qilian Mountains, forming a down-faulted closed inland basin. The elevations of the outer basin reach 4000–5000 m, and the lower parts of the basin are at 2700–3200 m. There are 51 lakes in the Qaidam Basin. Of these, Keluke Lake is a freshwater lake, and seven of the lakes have brackish water. The others are all saline lakes, and six of them are playas, which are rich in salt mineral resources^18^.

**Figure 1:**
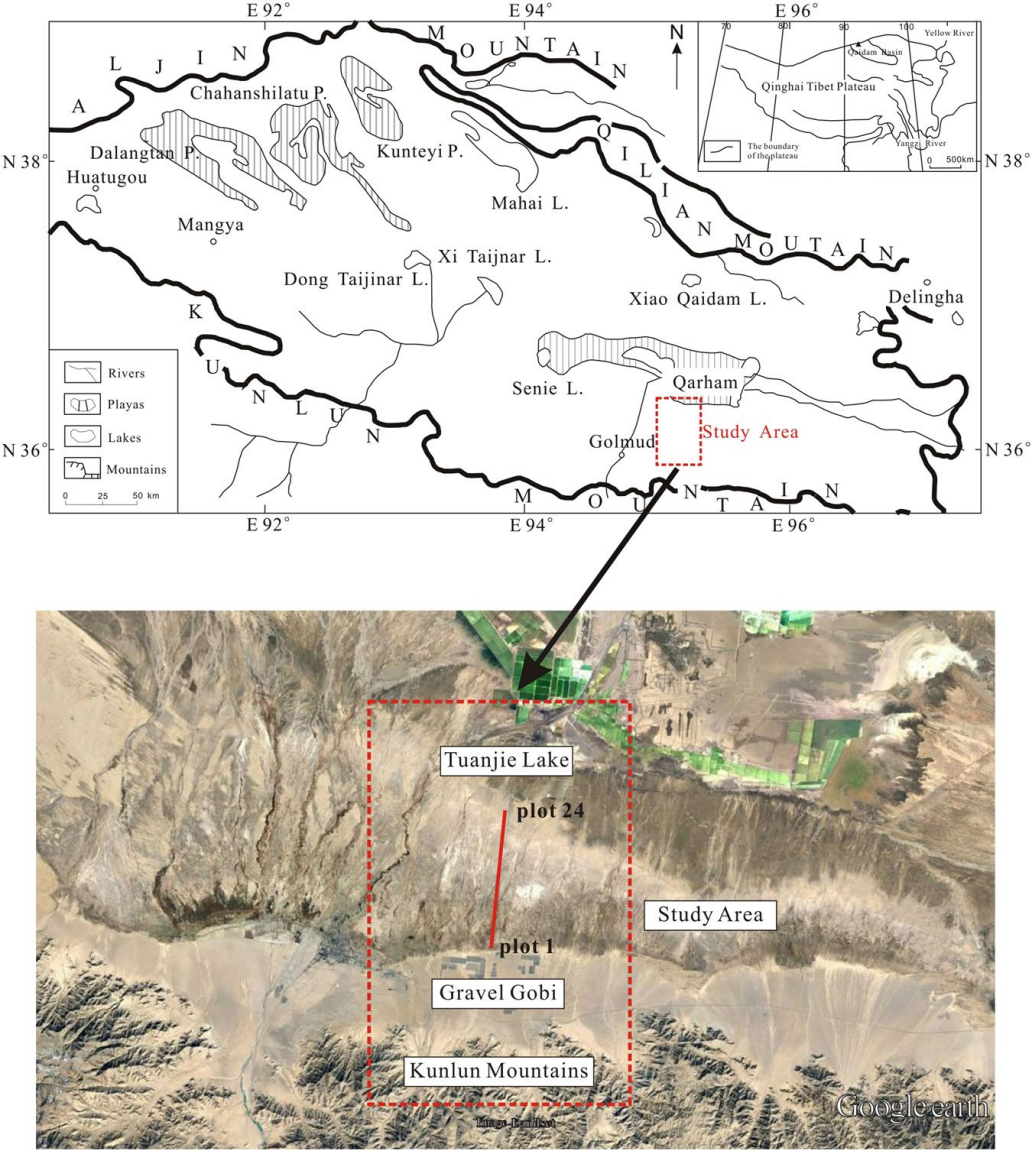
The distribution of saline lakes in the Qaidam Basin and the location map of the study site. Plots 1 to 24 are distributed from a gravel Gobi area to Tuanjie Lake. The upper part of the Fig. 1 was drawn by Xianjie Wang using Corel DRAW software (Corel DRAW X4 14.0.0.701), and the lower part was modified from map downloaded from Google Earth on May 2017 (Google Earth 7.1.5.1557).

The study site is located in the southern part of the Qaidam Basin, 22 km east of the city of Golmud. The site spans Tuanjie Lake to the diluvial plain on the north piedmont of the Kunlun Mountains. The plain (generally gravel Gobi or sand), which is mainly on the edge of alluvial plains, has some areas of fine soil and fluvial plain. Its dimensions range from approximately N36°37′–N36°25′, E95°11′10.5″–E95°11′13.6″, with an altitude of approximately 2750 m (Fig. 1).

Tuanjie Lake is formed atop the surrounding salt marshes of Qarhan Salt Lake and recharged by river water or groundwater. Phreatic water in the study site is close to the surface or overflowing, and the plant communities are dominated by *Tamarix* and salt-tolerant hygrophytes. The plant community structures are simple, having relatively few species. The predominant soil type is solonchak, according to the Food and Agriculture Organization classification^19^.

The climate of the area is temperate and arid with a mean annual precipitation of 28.1 mm, but a mean annual evapotranspiration of 3456 mm^20^. The average temperature is 5.2 °C. The total annual sunshine duration is long, exceeding 3100 h. The total solar radiation is 700 kJ/cm^2^, and the average wind speed is 4.3 m/s^21^. In summary, the climate of the basin can be described as cold, dry and windy, with a large diurnal temperature difference, long sunshine duration and strong solar radiation.

Since the extreme harsh environment condition for the vegetation, the ecological environments here are fragile. The objective of this study was to investigate the relationship between soil and vegetation distribution, and establish a natural vegetation distribution model for saline lakes on an inland plateau. This model allows detection of environmental changes based on vegetation observations, which will help prevent environmental impacts and contribute to the management and conservation of this area^22^. Studies including both quantitative and qualitative environmental data from the area are needed to restore and preserve it. They can also provide basic data for the development of agriculture in the area, particularly for the saline lake agriculture.

## Results

### Vegetation characteristics

The plant types in the study site included shrubs and herbs. Shrub species included *Tamarix*, *Nitraria sibirica*, and *Lycium ruthenicum*. Herb species included *Phragmites australis*, *Leymus secalinus*, *Apocynum venetum*, *Salicornia europaea*, *Saussurea salsa*, *Kalidium gracile*, *Sphaerophysa salsula*, and *Glaux maritima*.The coverage of the vegetation is shown in Table 1. Spots 1 and 2 were located in the gravel Gobi vegetation on the diluvial clinoplain at the north piedmont of the Kunlun Mountains, and only a few reeds were distributed sparsely. With the lower water table, the surface soil transitioned to salt crust, where the dominant species were *Tamarix*, *Lycium ruthenicum*, *Apocynum venetum*, and *Phragmites australis* (spots 3 to 6). From spots 7 to 11, the soil moisture increased, and some salt-tolerance hygrophytes, such as *Leymus secalinus*, *Glaux maritima*, and *Triglochin maritimum* were present. Close to the playa centre, the vegetation was predominantly *Tamarix*, *Phragmites australis*, *Apocynum venetum*, *Saussurea salsa*, and *Nitraria sibirica*. In the lowest terrain of the playa, the only plants were *Phragmites australis*, and the coverage was very low (spots 22 and 23). The northern part next to spot 23 was a bare flat area covered with extensive salt crust (spot 24).

**Table 1:** Coverage of 10 species from 24 quadrats taken in the Tuanjie Lake salt marshes.

	*Phragmites australis*	*Apocynum venetum*	*Lycium ruthenicum*	*Tamarix*	*Leymus secalinus*	Saliconia europaea	*Saussurea salsa*	Kalidium gracile	*Sphaerophysa salsula*	*Glaux maritime*
1	—	—	—	—	—	—	—	—	—	—
2	2	—	—	—	—	—	—	—	—	—
3	5	6	—	—	—	—	—	—	—	—
4	4	2	5	—	—	—	—	—	—	—
5	2	5	4	—	—	—	—	—	—	—
6	4	4	2	4	—	—	—	—	—	—
7	4	—	—	—	7	4	—	—	—	—
8	2	—	—	—	2	1	—	—	—	1
9	6	—	—	—	6	—	4	—	—	—
10	5	—	—	—	—	2	7	—	—	—
11	5	—	—	—	5	—	4	—	4	—
12	1	—	—	—	—	—	—	—	—	—
13	4	—	—	—	—	—	—	—	—	—
14	2	—	—	2	—	—	—	—	—	—
15	2	5	—	—	—	—	—	—	—	—
16	2	—	—	—	—	—	1	—	—	—
17	4	5	—	—	—	—	—	—	—	—
18	4	1	—	5	—	—	—	—	—	—
19	1	1	—	5	—	—	—	—	—	—
20	1	4	—	—	—	—	—	—	—	—
21	1	4	—	2	—	—	—	4	—	—
22	1	—	—	—	—	—	—	—	—	—
23	1	—	—	—	—	—	—	—	—	—
24	—	—	—	—	—	—	—	—	—	—

Notes: Cover scale:—indicates 0% cover; 1 indicates <1% cover; 2 indicates 1–5% cover; 3 indicates 5% cover; 4 indicates 5–12.5% cover; 5 indicates 12.5–25% cover; 6 indicates 25–50% cover; 7 indicates 50–75% cover; 8 indicates >75% cover. Species coverage = π × (crown width/2)^2^ × quantity/25.

In the study site, *Phragmites australis* was distributed widely, with high coverage in almost every sampling spot, followed by *Apocynum venetum, Tamarix*, and *Lycium ruthenicum*. These plants mainly occurred in spots 3 to 6 and spots 14 to 21. *Saussurea salsa, Leymus secalinus, Salicornia europaea, Sphaerophysa salsula*, and *Glaux maritima* were concentrated in spots 7 to 11.

### Spatial variations in the physical and chemical parameters of the soil

**Figure 2:**
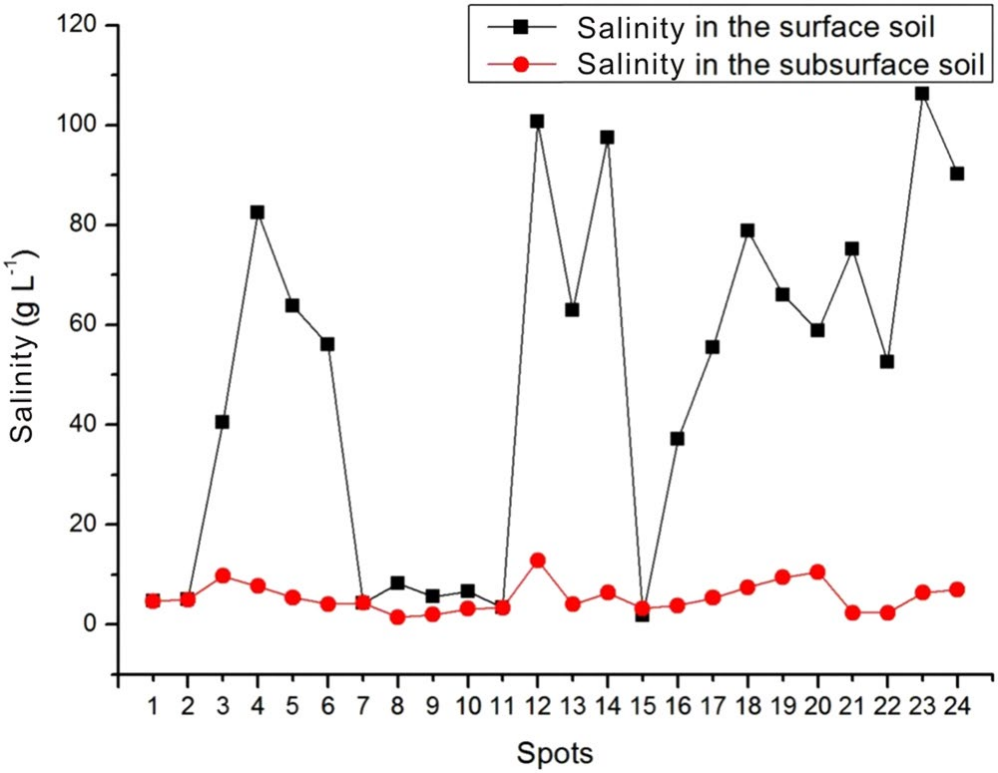
The distribution of soil salinity.

Most soils in the study area were sandy and loamy. The salinity in the surface layer was greater than that in the deeper layers. The surface layer of most of the soils was a white salt crust, with a thickness of approximately 2–15 cm. However, the subsurface soil was mainly salty brown sand. The pH of the soils in the study site ranged from 8.0 to 9.0, indicating that the soils are alkaline. The physical and chemical characteristics were measured in the laboratory. The results showed that the salinity in most surface soils exceeded 92.875 g/kg, with a maximum value of 265.75 g/kg at spot 23. The salinity of all of the subsurface soil samples was below 32.2 g/kg; a minimum value of 3.608 g/kg occurred at spot10. These results suggest that there is a strong salt gradient in the vertical direction. Laterally, from the border of the north piedmont of the Kunlun Mountains to the Tuanjie Lake salt marshes, the surface soil had fluctuating salinity, but the salinity of the subsurface soil remained relatively constant (Fig. 2).

**Figure 3:**
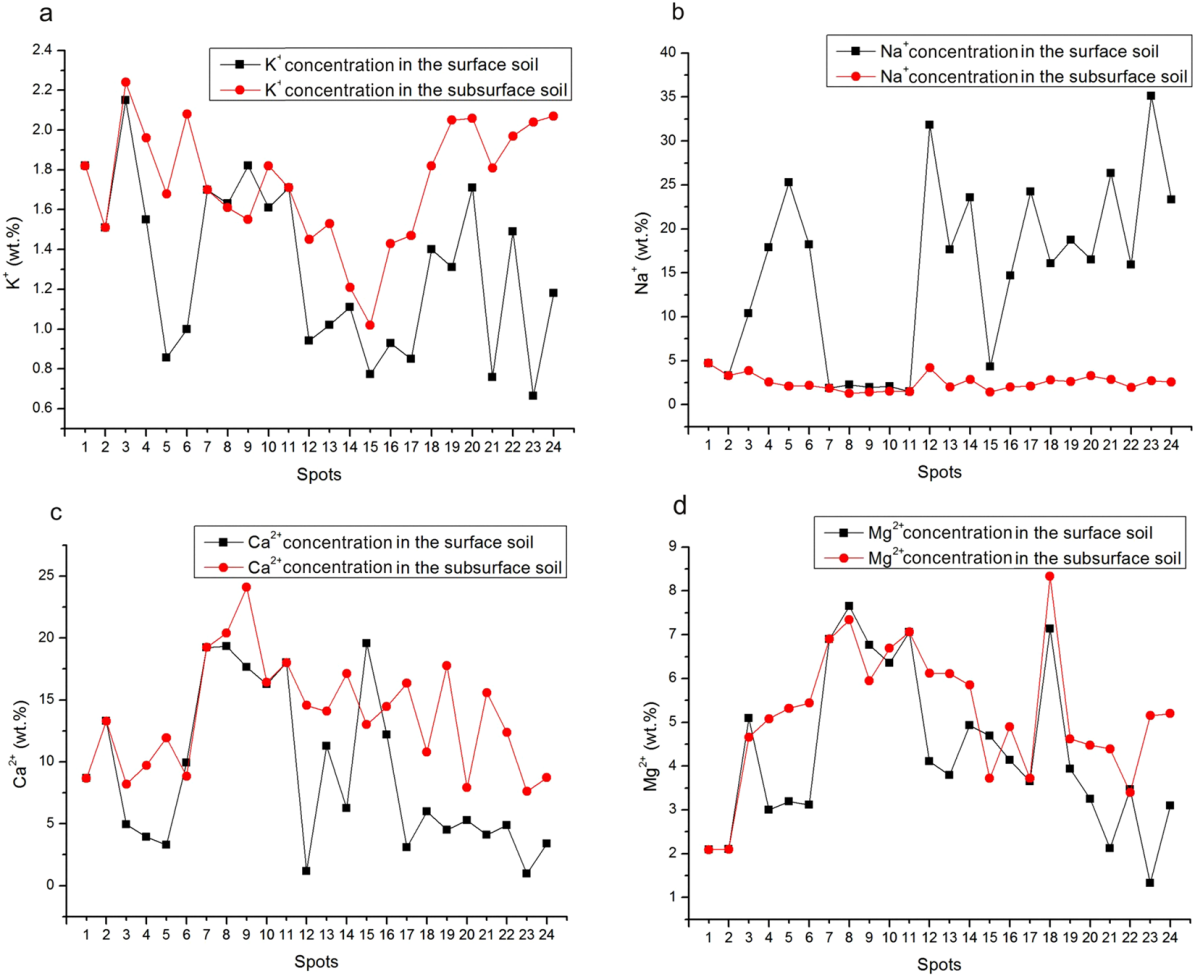
The distributions of the main soil cations. (**a**) K^+^ concentration; (**b**) Na^+^ concentration; (**c**) Ca^2+^ concentration; (**d**) Mg^2+^ concentration.

**Figure 4:**
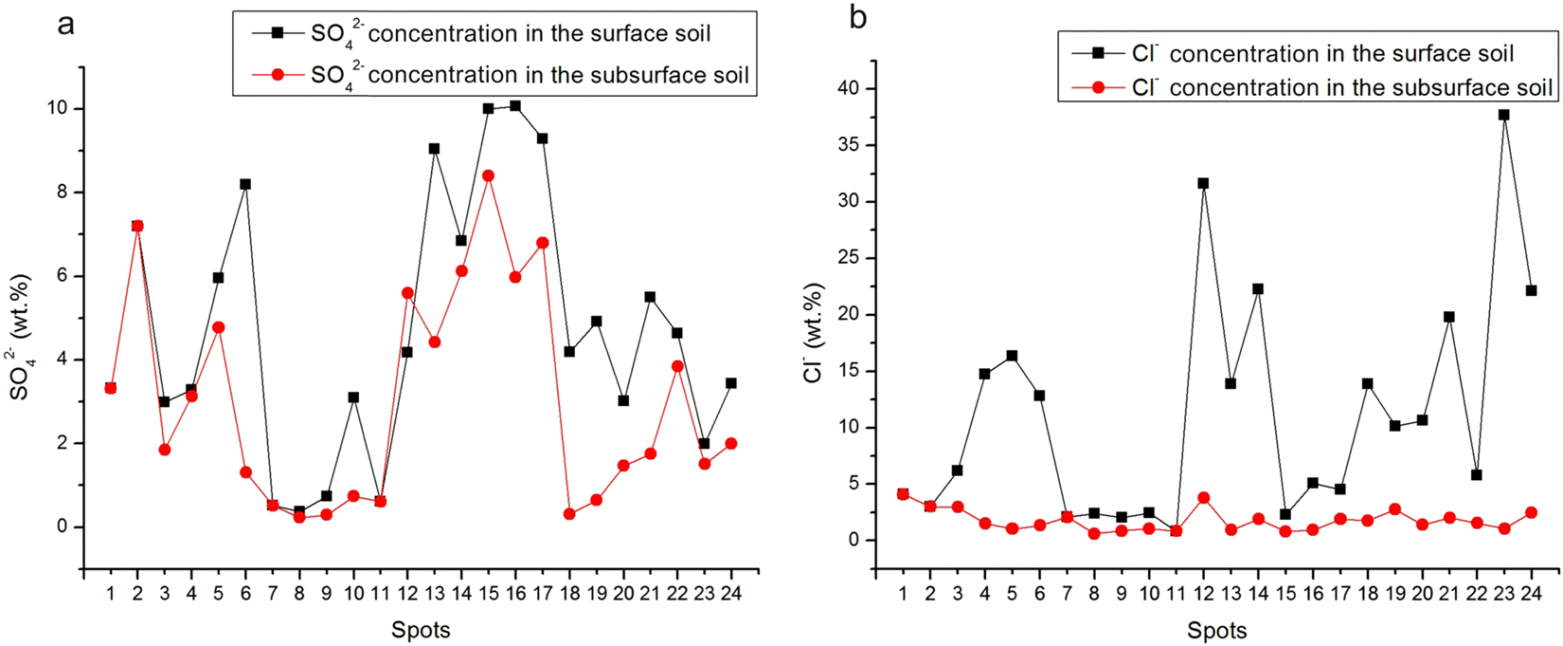
The distributions of the main soil anions. (**a**) SO_4_^2−^ concentration; (**b**) Cl^−^ concentration.

Except for spots 1, 2 and 7–11, the Na^+^ concentration of the soil decreased greatly with depth. In contrast, the K^+^, Ca^2+^, and Mg^2+^ concentrations increased with depth in most of the sampled spots. The lateral distribution of the Na^+^ concentration was similar to that of the salinity. The concentrations of Mg^2+^ and Ca^2+^ first increased and then decreased, and the K^+^ concentration was relatively uniform (Fig. 3). The concentrations of Cl^−^ and SO_4_^2−^ were all higher in the surface than in the subsurface layers (Fig. 4). NaCl tended to be concentrated in the surface layer. In subsurface soil, the major soluble cation was Ca^2+^, and the major anions were Cl^−^ and SO_4_^2−^.

Ion concentrations in the soils from spots 7 to 11 were relatively low and varied less than those from the other sampling spots; the ion concentrations did not vary much with depth. The low salinity could have been due to the dilution of salt in wet soils that were located in topographic lows with a shallow groundwater level. Furthermore, relatively weak surface accumulation of salt due to low capillary action could account for the lack of substantial vertical variation. Other key features of the soils from spots 7 to 11 include high concentrations of Ca^2+^ and Mg^2+^ and a Cl^−^ concentration that was higher than that of SO_4_^2−^.

### The analysis of relationship between soil and vegetation by principal component analysis

**Figure 5:**
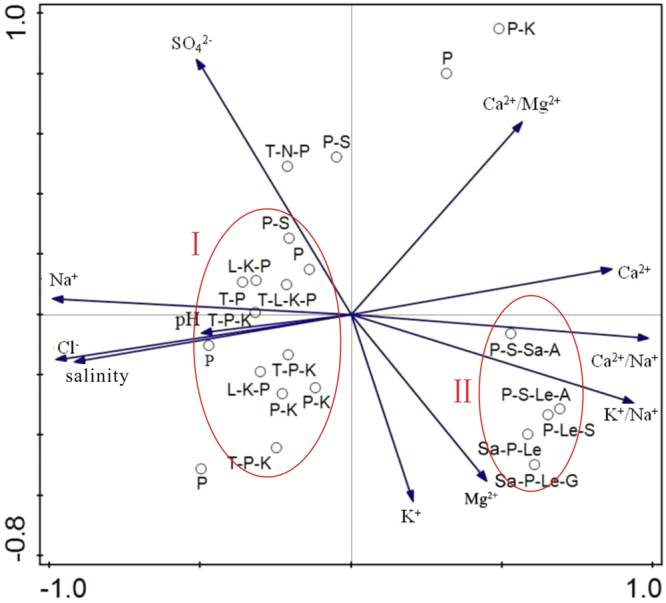
Results of the PCA performed on the sample data. P: *Phragmites australis* P-K: *Phragmites australis—Apocynum venetum* P-S: *Phragmites australis—Saussurea salsa* T-P: *Tamarix—Phragmites australis* L-K-P: *Lycium ruthenicum—Apocynum venetum—Phragmites australis* T-P-K: *Tamarix—Phragmites australis—Apocynum venetum* T-N-P: *Tamarix—Nitraria sibirica—Phragmites australis* Sa-P-Le: *Saliconia europaea—Phragmites australis—Leymus secalinus* P-Le-Sa: *Phragmites australis—Leymus secalinus—Saliconia europaea* T-L-K-P: *Tamarix -Lycium ruthenicum—Apocynum venetum—Phragmites australis* Sa-P-Le-G: *Saliconia europaea—Phragmites australis—Leymus secalinus—Glaux maritima* P-S-Sa-A: *Phragmites australis—Saussurea salsa—Saliconia europaea –Asparagus* L. P-S-Le-A: *Phragmites australis—Saussurea salsa—Leymus secalinus—Asparagus* L.

To examine the plant-soil relationships, principal component analysis (PCA) was performed using the CANOCO 5 suite^23^. Dots represent the vegetation communities of the 24 sampling spots. The PCA results showed that the sum of the eigenvalues of the first and second axes was equal to 94% of the total sum of eigenvalues for all axes; as they account for most of the variance, we only considered the first and second axes in further analysis (Fig. 5).

PCA axis 1, with an eigenvalue of 0.83, was best correlated with Na^+^, Cl^−^, salinity, Ca^2+^, and the Ca^2+^/Na^+^ and K^+^/Na^+^ ratios. On the positive side of this axis are species that grow in soils with low concentrations of salt and Na^+^ and high concentrations of Ca^2+^ and K^+^. In contrast, the negative side includes species that grow best in soils with high salinity, high concentrations of Na^+^ and Cl^−^, and a low concentration of Ca^2+^. The second axis, with an eigenvalue of 0.11, was primarily related to the SO_4_^2−^ and K^+^ concentrations. Since its eigenvalue was small, so it had small impact on the distribution of vegetation. As shown in Fig. 5, the vegetation at the study site can be divided into two categories: I) salt-tolerant vegetation communities including *Phragmites australis*—*Apocynum venetum*, *Tamarix* -*Phragmites australis*—*Apocynum venetum*, *Lycium ruthenicum*—*Phragmites australis*—*Apocynum venetum*, *Tamarix*—*Lycium ruthenicum* -*Phragmites australis*—*Apocynum venetum*, and II) weakly salt-tolerant hygrophytes, such as *Leymus secalinus*, *Glaux maritima*, and *Asparagus* L. The species of type I were influenced by Na^+^, Cl^−^, and the salinity. The soil corresponding to these vegetation communities had higher salinity and Na^+^ concentrations and lower Ca^2+^and K^+^ concentrations. The species of type II were mainly controlled by the Ca^2+^/Na^+^ and K^+^/Na^+^ ratios. In these communities, the soil salinity and Na^+^ concentrations were low, and the Ca^2+^ concentration was high.

## Discussion

### Spatiotemporal gradients of saline soil conditions in the Qaidam Basin

The Qaidam Basin is an inland arid plateau basin located in the north-eastern part of the Qinghai-Tibetan Plateau. The climate of coastal marshes alternates between periods of rainfall periods and drought. Salt is leached to the deepest soil horizons during the rainfall periods and rises to shallower horizons during the drought periods, resulting in the accumulation of salt near the surface^6,12^. In contrast to the coastal marshes, the Qaidam Basin is surrounded by mountains that prevent warm, south-westerly air from entering the basin’s interior, resulting in minimal precipitation^24^. In addition, the high elevation together with strong sunshine and solar radiation, result in the long-term accumulation of salt at the study site. The surface soil salinity is extremely high, and the seasonal variability of soil salinity is relatively low. Maritime salt marshes are periodically flooded by seawater^25^; thus, the contents of Na^+^ and Cl^−^ are far higher than those of other ions. However, in inland areas, salt marshes are dominated by chlorides, sulphates, carbonates or bicarbonates. At the study site, the salt is mainly derived from leaching of rock salt. The salt marshes are dominated by chloride-type and sulphate-type soils.

The high gravel content in soil samples collected from the desert zone of the north piedmont of the Kunlun Mountains, together with the low groundwater level, would create a weak capillary effect, leading to the low soil salinity observed at spots 1 and 2. A higher groundwater level and soil sand content would enhance the capillary effect, resulting in increased soil salinity and surface salt accumulation (spots 3 to 6). At spots 7 to 11, the groundwater was near the surface in the lower terrain, and the soil salinity was very low. Close to the playa centre, the soil salinity was higher, reaching its maximum value near the centre of the playa lake (spots 22–24). Horizontally, the surface soil salinity was varied.

In the study area, the surface salt crust is very thick. The main factors leading to the formation of these crusts are aridity, high evaporation and salty groundwater^26^. When studying salt marshes in south-eastern Spain, Álvarez Rogel noted that with increased salinity, the relative percentages of Ca^2+^ and K^+^ decreased, leading^26^ to an imbalance in favour of the most toxic cations, such as Na^+^ and Mg^2+^ ^1^. Similar results were also obtained in the present study. At the study site, moist soil had low salinity and a high concentration of Na^+^ concentration and Ca^2+^. Soil with less moisture had high salinity, with lower Ca^2+^ and higher Na^+^ concentration, though the distributions of the K^+^ and Mg^2+^ concentrations were not remarkable.

Many factors influenced the distribution of ions in the soil. For example, cations are absorbed by the soil’s exchange complexes in the following sequence: Ca^2+^, Mg^2+^, K^+^, and Na^+^. Thus, during wet periods, divalent cations are more likely to be fixed in the surface horizons, increasing the Ca^2+^/Na^+^ and Ca^2+^/Mg^2+^ ratios. However, salts such as NaCl and MgCl_2_ are highly soluble and may be more mobile in the soil profile. In addition, changes in the concentration of NaCl affect the solubility of other salts^6^.

### Relationships between plant distributions and soils

In the study site, harsh climatic conditions and the unique geographical location cause considerable environmental stresses that affect vegetation growth; therefore, the soil salinity is an important factor affecting plant zonation. This result is consistent with previous studies that found that soil salinity was the decisive factor, especially in inland salt marshes^1,6,12–16^. However, not all ions increased with soil salinity. Soil salinity affects the overall vegetation distribution pattern, but specific ion concentrations or ion ratios can be further used to evaluate the relationship between soil and vegetation types^27^.

Table 2 shows characteristics of the sampling spots dominated by *Apocynum venetum, Lycium ruthenicum*, *Saussurea salsa*, and *Tamarix*, including the soil salinity and ion ratio. *Saussurea salsa* grew mostly in soil with low salinity and high moisture, such as a low-humidity river bank, the lowland saline land at the edge of a saline lake, saline sand soil, or swamp meadow. The K^+^/Na^+^ ratio (0.96), Ca^2+^/Na^+^ ratio (9.13) and Ca^2+^/Mg^2+^ ratio (2.51) in the soil dominated by *Saussurea salsa* were high. In other words, the K^+^ and Ca^2+^ concentrations were high, while the Na^+^ and Mg^2+^ contrations were low. *Saussurea salsa* can also grow in severely saline soil environments; however, the plants are short, and the coverage is low.

**Table 2:** The influence of the soil ion ratio on plant distribution.

	Salinity	K^+^/Na^+^	Ca^2+^/Na^+^	Ca^2+^/Mg^2+^
*Apocynum venetum*	25.11	0.31	0.92	1.35
*Lycium ruthenicum*	45.06	0.17	0.67	1.69
*Saussurea salsa*	4.92	0.96	9.13	2.51
*Tamarix*	37.67	0.16	1.04	2.60

*Apocynum venetum, Tamarix* and *Lycium ruthenicum* grew in soil with high salinity. Compared to the other two salt-tolerant plants, *Lycium ruthenicum* favoured soils dominated by NaCl that had low K^+^/Na^+^ and Ca^2+^/Na^+^ ratios of 0.17 and 0.67, respectively. Thus, the Na^+^ concentration was high, while the K^+^ and Ca^2+^ concentrations were relatively low.

*Apocynum venetum* also grew in soil dominated by NaCl, as well as sulphate-type soil. The Ca^2+^/Mg^2+^ ratio (1.35) was low, while the Mg^2+^ concentrations were high. The growth environment of *Lycium ruthenicum* was limited; it only appeared in spots 4 to 6. In contrast, *Apocynum venetum* was distributed in high salinity areas, showing its strong adaptability to hypersalinity.

*Tamarix* is salt-secreting plant that can grow in saline soil. *Tamarix* can excrete salt absorbed into its body through salt-secreting pores. The salt in the litter often adheres into blocks of sand to form a fixed sandbag^28,29^. Therefore, *Tamarix* was the typical vegetation in soils with high surface soil salinity, low K^+^/Na^+^ (0.16) and high Ca^2+^/Mg^2+^ (2.60). *Tamarix* preferred soils with lower magnesium concentrations compared to the other species.

Unlike soils in the coastal area of northern China, which have low salinity and high moisture content^30^, soils in the study area are dry and have high salinity. *Phragmites australis* is distributed widely in the Tuanjie Lake playa, and it is present at almost every sampling point. *Phragmites australis* is resistant to extreme environmental factors, such as cold, drought, salt, wind, and preferred to high-humidity environment. Some scholars have noted that succulent halophytes seem to be the first colonizers of bare soil^1^, but in this study site, the most salt-tolerant plant species was *Phragmites australis*.

### Vegetation distribution model of saline lakes on an inland plateau

**Figure 6:**
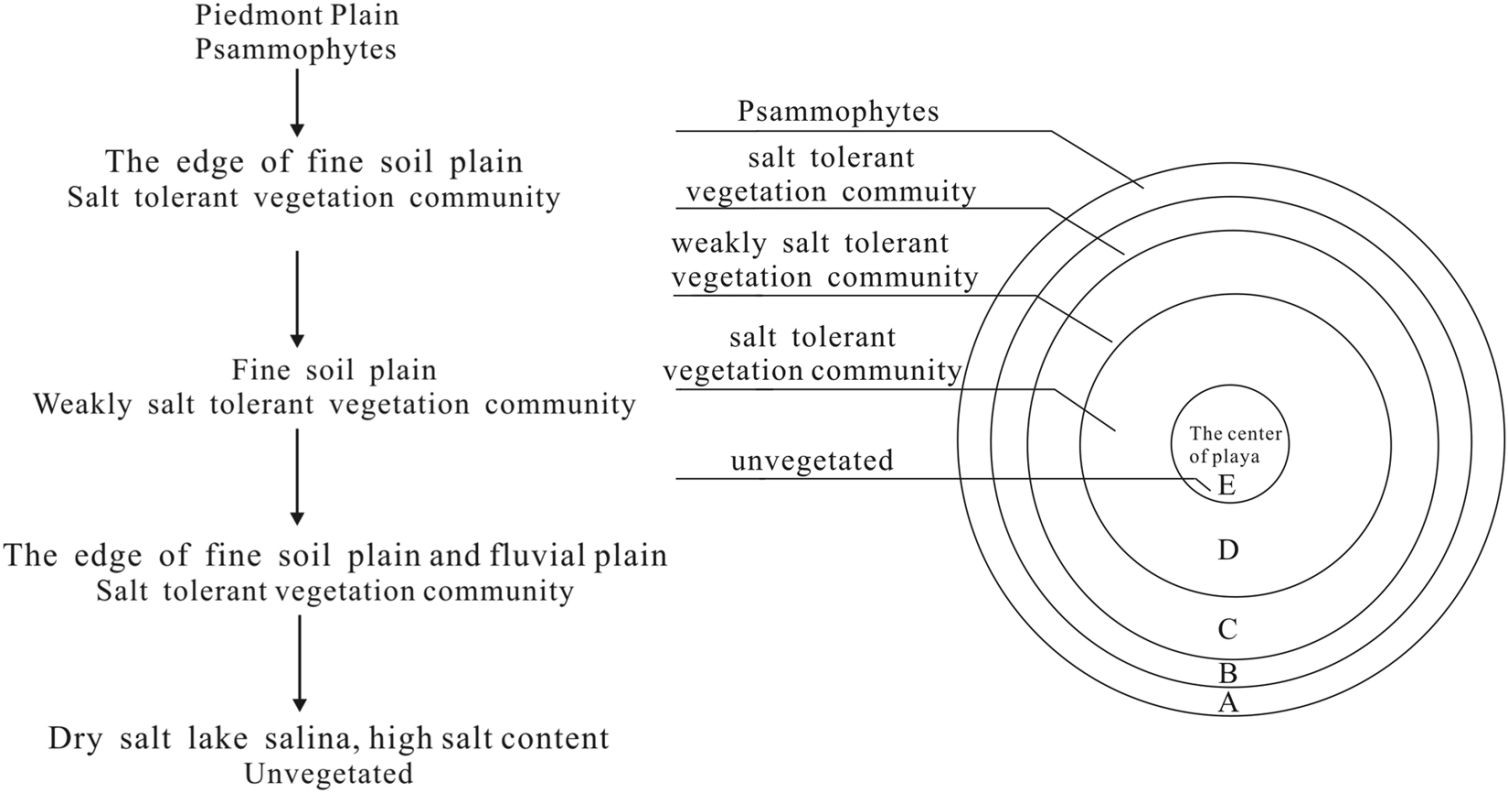
The vegetation is zoned distributed with the saline lake or playa at the centre.

Based on the results of this study and the conclusions of previous literature, a vegetation distribution model of saline lakes on an inland plateau was established. The terrain from the surrounding mountains to the centre of the basin includes mountains, Gobi Desert areas, sand dunes, fine soil plains, swamps and saline lakes. The vegetation is distributed in five zones around the saline lakes or playas at the centre (Fig. 6).

The Qaidam Basin water resources mainly come from meltwater and precipitation in mountainous areas. The mountains around the basin are the sources of numerous rivers, but after those rivers flow through the edge of the alluvial fan, most runoff enters the ground. Groundwater is deep and difficult for vegetation to access, resulting in the formation of Gobi and other desert landscapes. While the groundwater level of the alluvial fan increases, the sparse vegetation begins to appear. The vegetation type is mainly psammophytes, the soil gravel content is high, and the organic matter and salinity are relatively low (Area A).

With the increase in the groundwater level, the soil sand content also increases; the salinity and Na^+^ concentrations are high; and the Ca^2+^ concentration is relatively low. The surface soils have a salt crust, which may be very thick. This area is dominated by the salt-tolerant vegetation community, which includes *Tamarix, Nitraria sibirica, Phragmites australis, Apocynum venetum*, and *Lycium ruthenicum*. The roots of these salt-tolerant species can penetrate the surface of the heavy salt layer and absorb water and nutrients from the subsurface soil^31^ (Area B).

In the fine soil plains, the soil texture is silt or clay and the groundwater level is close to the surface. The main vegetation type is weakly salt-tolerant hygrophytes, such as *Triglochin maritimum, Glaux maritima*, and *Leymus secalinus*. The soil has low salinity and Na^+^ concentrations and a high Ca^2+^ concentration and water content. Plant growth pressure in this environment is low. The soil type is primarily loam and clay. The vegetation abundance and coverage are high, which creates a good pastoral area (Area C).

With increased proximity to the centre of a saline lake, the groundwater level decreases at the edge of the fine soil plain and lacustrine plain areas of dry saline lake; soil salinity and Na^+^ concentration increase. The surface soil often forms a salt crust ranging from a few to over 10 cm thick. Throughout the zone, the vegetation associations are gradually dominated by halophytes, driven by salinity (Area D). The playa centre is bare saline land with no vegetation (Area E).

The model also reflects the relationship between the vegetation types and soil environment. For instance, increased coverage of *Triglochin maritimum, Glaux maritima, Leymus secalinus* and *Saussurea salsa* indicates a high water content, low salinity, high K^+^ and Ca^2+^ concentrations and low Na^+^ and Mg^2+^ concentrations. Increased coverage of *Tamarix, Lycium ruthenicum*, and *Apocynum venetum* indicates a low water content, high salinity, low K^+^ and Ca^2+^ concentrations and high Na^+^ and Mg^2+^ concentrations.

Based on the above description, the idealized distribution of vegetation in an inland plateau saline lake is zone. However, this zoned pattern is often disrupted by water systems and geological structures such as in the Tsagaan Us-Wutumeiren Basin and the Delingha Basin. Therefore, it is necessary to study the actual conditions of the vegetation and soil in each saline lake.

## Materials and Methods

### Collection of soil samples and analysis

Field investigations were carried out from August to September 2015, and samples were collected from the gravel Gobi areas on the diluvial clinoplain in the north piedmont of the Kunlun Mountains to the Tuanjie Lake salt marshes (Fig. 1). There were 24 sampling sites within the 22-km transect. The vegetation data were recorded using 5 m × 5 m quadrats. The relative plant height, crown width and quantity of each species were measured, and the coverage of each species was calculated. Soil samples were taken layer by layer, and the sampling depth was 0–40 cm. Because sample plots 1, 2, 7 and 11 were not distinct, soil samples were collected from one layer. In the remaining sampling plots, samples were collected from two layers. A total of 44 soil samples were collected.

All of the soil samples collected in the field were analysed in the laboratory. The samples were dried at 65 °C and passed through a 2-mm sieve to remove coarse fragments before laboratory analysis. Salinity was measured in a 1:2.5 soil water extraction using an electrical conductivity method, following an established procedure^32^. The concentrations of K^+^, Na^+^, Ca^2+^, Mg^2+^, Cl^−^, and SO_4_^2−^ were measured using a Perform X (Thermo Fisher Inc., U.S.A.) X-ray fluorescence spectrometer (XRF; effective diameter = 25 mm).

### Statistical analysis

Principal component analysis (PCA) was performed using the programme CANOCO 5 to evaluate plant-soil relationships. When the cumulative variance of the analysis result was close to or greater than 80%, the PCA was considered useful. The results can be presented in ordination diagrams, which include arrows that represent environmental variables and points that represent species. A shorter vertical distance between a point and environmental variables indicates that the environmental variables have a greater effect on the distribution of vegetation^20^.
